# Multiple Mechanisms Contribute to Leakiness of a Frameshift Mutation in Canine Cone-Rod Dystrophy

**DOI:** 10.1371/journal.pone.0051598

**Published:** 2012-12-12

**Authors:** Keiko Miyadera, Ian Brierley, Jesús Aguirre-Hernández, Cathryn S. Mellersh, David R. Sargan

**Affiliations:** 1 Department of Veterinary Medicine, University of Cambridge, Cambridge, United Kingdom; 2 Virology Division, Department of Pathology, University of Cambridge, Cambridge, United Kingdom; 3 Canine Genetics Section, Animal Health Trust, Newmarket, United Kingdom; VIB & Katholieke Universiteit Leuven, Belgium

## Abstract

Mutations in *RPGRIP1* are associated with early onset retinal degenerations in humans and dogs. Dogs homozygous for a 44 bp insertion including a polyA_29_ tract potentially leading to premature truncation of the protein, show cone rod degeneration. This is rapid and blinding in a colony of dogs in which the mutation was characterised but in dogs with the same mutation in the pet population there is very variable disease severity and rate of progression.

**Objective:**

We hypothesized that this variability must be associated with leakiness of the *RPGRIP1* mutation, allowing continued RPGRIP1 production. The study was designed to discover mechanisms that might allow such leakiness.

**Methods:**

We analysed alternate start sites and splicing of *RPGRIP1* transcripts; variability of polyA_n_ length in the insertion and slippage at polyA_n_ during transcription/translation.

**Results and Significance:**

We observed a low rate of use of alternative start codons having potential to allow forms of transcript not including the insertion, with the possibility of encoding truncated functional RPGRIP1 protein isoforms. Complex alternative splicing was observed, but did not increase this potential. Variable polyA_n_ length was confirmed in DNA from different *RPGRIP1* ^−/−^ dogs, yet polyA_n_ variability did not correspond with the clinical phenotypes and no individual was found that carried a polyA_n_ tract capable of encoding an in-frame variant. Remarkably though, in luciferase reporter gene assays, out-of-frame inserts still allowed downstream reporter gene expression at some 40% of the efficiency of in-frame controls. This indicates a major role of transcriptional or translational frameshifting in *RPGRIP1* expression. The known slippage of reverse transcriptases as well as RNA polymerases and thermostable DNA polymerases on oligoA homopolymers meant that we could not distinguish whether the majority of slippage was transcriptional or translational. This leakiness at the mutation site may allow escape from severe effects of the mutation for some dogs.

## Introduction

Leber congenital amaurosis type 6 (LCA6) is a retinal dystrophy causing profound vision loss, often from birth, nystagmus and sometimes unrecordable electroretinogram (ERG). It is associated with homozygous (or compound heterozygous) nonsense or missense mutations in the Retinitis Pigmentosa GTPase Regulator Interacting Protein 1 (*RPGRIP1*) [1], [2], [3]. Degeneration is observed in both rod and cone photoreceptors. In common with most forms of human retinal degeneration, there is heterogeneity in onset and progression. Although patients with homozygous null mutations show profound loss of retinal function from early childhood, for some patients with missense mutations, vision is retained at birth but progressively lost in the first two decades (human cone-rod dystrophy type 13, CORD13) [4].

Cone-rod dystrophy 1 (*cord1*) in the miniature long haired dachshund (MLHD) is considered as orthologous to the human condition. In work with a colony of dogs the disease has been described as early onset, with ERG deficits measurable within six weeks of birth, and complete loss of ERG by 40 weeks. In this colony, there was complete functional blindness before two years of age [5], [6], [7]. The disease in the colony segregated as autosomal recessive, showing association with an exonic insertion mutation in *RPGRIP1* [8]. This mutation is a polyA tract insertion of 29 nucleotides flanked by a 15 bp duplication. This was expected to lead to a change of reading frame and a stop codon early in the following exon of the *RPGRIP1* gene, and thus truncation of the encoded protein.

Homozygosity for the insertion (*RPGRIP1* ^−/−^) is relatively common in the pet population in MLHD and in several other dog breeds [5], [9]. The age of clinical onset in the *RPGRIP1^−/−^* MLHD pet population is highly variable with about half of such dogs having severe disease before three years of age and the remainder having delayed and variable onset and slower progression (onset 5–15 y, with some animals showing no obvious clinical signs in their lifetimes) [9]. In some beagles, a ∼44 bp polyA tract has been found at the same position, flanked by a 15 bp duplication identical to that seen in MLHD. In beagles homozygous for this insertion variant (*RPGRIP1* ^−L/−L^), partial loss of neuro-retinal function is measurable at the level of ERG but seems to remain subclinical in all animals. In particular, *RPGRIP1* ^−L/−L^ beagles may suffer severe loss of cone-mediated ERG with moderate reduction of rod-mediated ERG, without fundus or behavioural abnormalities [9]. The polyA tract is ∼15 bp longer in beagles than in MLHD and is likely to lead to the same frameshift and nonsense mutation.

We were puzzled by the presence of the homozygous insertion mutation in clinically normal dogs, although this causes a nonsense mutation in what in humans and mice is an essential gene for vision. The simplest prediction would be that such a mutation would knock out protein production by *RPGRIP1*, giving a homogeneous severe phenotype. The incomplete association between insertion and disease had previously led us to fine map the originally published (14 Mb) confidence interval [8], to see if there is closer association of the disease phenotype with any part of the locus other than *RPGRIP1*. This fine mapping across affected breeds showed that the *RPGRIP1* insertion is very closely associated with the disease in MLHD whilst the confidence interval has been reduced to a maximum of 0.51 Mb containing 17 genes [9]. None of the other genes are obvious candidates for mutations causing non-syndromic retinal degeneration. Sequencing of DNA selected by DNA capture from across the confidence interval containing the *cord1* associated locus has not confirmed any other polymorphisms likely to be of significance to the disease (Table S1).

But the unexpected result is not that some *RPGRIP1* ^−/−^ dogs lose vision, it is that some dogs do not. In this paper, we look at possible mechanisms by which *RPGRIP1* ^−/−^ or *RPGRIP1* ^−L/−L^in dogs could be leaky, allowing maintained retinal function. We test the following hypotheses: that 1) retinal mRNA includes functional transcripts that bypass the insertion mutation through use of alternative transcription start sites or alternate splicing; that 2) slippage by DNA polymerases on the polyA tract means that there are genomic variants in insertion length in different individuals of the MLHD population including dogs with variants in which the insertion length is divisible by three. In these dogs, transcriptional read-through of the insertion gives a functional RPGRIP1 protein, leading to less severe disease; or that 3) slippage by RNA polymerase II or frameshifting by ribosomes on the polyA tract may restore the reading frame in some transcripts even though the insert length would normally cause a change of reading frame.

## Results

### Identification of Multiple Canine *RPGRIP1* mRNA Isoforms

**Figure 1:**
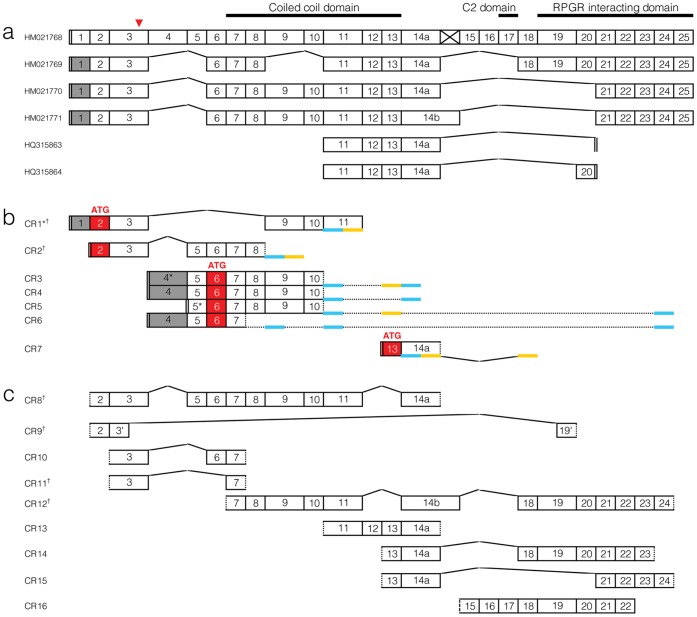
Partial exon structure of canine *RPGRIP1*. Canine *RPGRIP1* structure identified by Kuznetsova et al. [12] (a) and in this study by 5′ RACE (b) and RT-PCR (c). The red triangle points to the 44 bp insertion in exon 3 previously associated with *cord1* in a MLHD research colony [8]. The vertical double bar represents cDNA ends. Grey exon boxes indicate partially omitted sequences. Red exon boxes represent translation initiation site codon (ATG). (b) Blue and yellow underlines show location of primers used in RACE. †New transcript variants identified in the current study.

*RPGRIP1* is subject both to alternative splicing and to the use of internal promoter sites in human, murine and bovine retinas [2], [10], [11]. Kuznetsova et al. have described a group of canine retinal *RPGRIP1* isoforms (Figure 1a) [12]. To identify canine retinal *RPGRIP1* transcripts which had the potential to bypass the 44 bp insertion in exon 3, RNA extracted from clinically normal wildtype (*RPGRIP1* ^+/+^) canine retina was analysed. To identify the 5′ end of canine *RPGRIP1* transcripts, sets of reverse primers in different regions of *RPGRIP1* were used in 5′ RACE. Each reaction resulted in multiple products of different sizes, which were separated and sequenced. Clusters of 5′ ends were identified upstream from alternative translation initiation sites in exons 2, 6 and 13 (Figure 1b). An upstream non-coding exon was identified in a proportion of transcripts, renumbering the exon containing the insertion site as exon 3. Transcripts with 5′ ends between exons 3 and 5 were expected to utilize the AUG codon in exon 6. The transcription start site prediction programme Promoter 2.0 [13] had a high likelihood prediction of a start site close to 21,331,560 bp (CFA15, CanFam2.0) in agreement with this finding. Isoform CR7 represented an ORF starting from the first ATG in exon 13. Although Promoter 2.0 had only a marginal transcription start prediction centred at 21,358,970 bp in exon 12, the more stringent promoter prediction programme Eponine [14] suggested a high probability transcription start site between exons 12 and 13 at positions 21,359,033-37 bp in agreement with the RACE findings.

RT-PCR of several overlapping fragments across *RPGRIP1* cDNA resulted in multiple products of different sizes from each primer set. By sequencing each product after separation by agarose gel electrophoresis, multiple known and new *RPGRIP1* splice variants were identified. Observed fragments are displayed individually as we do not have evidence as to how these assemble into full transcripts. In agreement with a previous study [12] (Figure 1a), alternative splicing at exons 3,14a, and 14b, leading to skipping of several neighbouring exons between exons 15 and 20 was confirmed (Figure 1c). Exon 3, which harbours the *RPGRIP1* insertion mutation, was contained in at least four distinct transcript variants but was absent in RACE products having 5′ cDNA ends downstream of exon 3. One RT-PCR product also omitted the insertion site (CR9 in Figure 1c). An open reading frame was maintained in isoform CR9 that would encode a highly truncated protein missing at least two likely functional domains (see discussion).

### qRT-PCR Measurement of Transcript Concentrations

**Figure 2:**
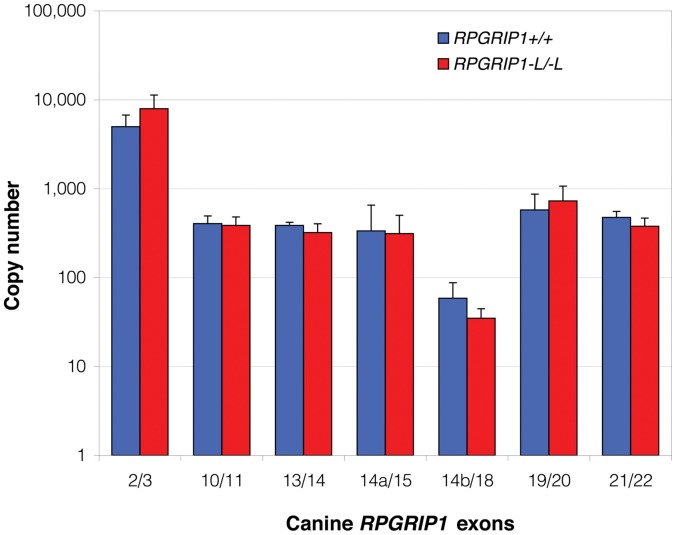
Quantitation of cDNA fragments of different exonic regions of *RPGRIP1*. Retinal cDNA populations were analysed by quantitative RT-PCR in beagles of *RPGRIP1* ^+/+^ (blue) and *RPGRIP1* ^−L/−L^ (red) genotypes. The absolute copy number of molecules in equal amounts of template cDNA is shown on a log scale. Each fragment was assayed in triplicate (technical replicates) and two replicate experiments and copy numbers of DNA molecules were calculated by comparison with control sequences cloned into plasmids.

Concentration of transcription products containing different parts of *RPGRIP1* was studied by qRT-PCR using retinal cDNA from *RPGRIP1* ^+/+^ and *RPGRIP1* ^−L/−L^ beagle dogs; both with no clinically observable visual dysfunction. In the retinal cDNA population, marked differences were observed in copy numbers between each exonic fragment. The transcript fragment encompassing exons 2 and 3 (exon 2/3) was the most abundant transcript in both *RPGRIP1* ^+/+^ and *RPGRIP1* ^−L/−L^ retina (Figure 2). Another five regions (exons 10/11, 13/14, 14a/15, 19/20, and 21/22) had transcript levels about ten-fold lower than exon 2/3. The transcript fragment with the lowest measured abundance was exon14b/18, an alternative transcript of exon 14a/15 with variant splice donor and acceptor sites. The relatively reduced level of transcripts containing exons 10/11 and 13/14 compared to exons 2/3, indicates that rather little use is made of the alternative transcription start sites in exons 6 and 13. The absolute level of *RPGRIP1* transcripts did not differ substantially between *RPGRIP1* ^+/+^ and *RPGRIP1* ^−L/−L^ retina. This finding was consistent both for randomly primed and Oligo dT primed cDNA populations (data not shown).

### Allelic Variation of the polyA Tract

#### 1. Relative sizing of PCR products

**Figure 3:**
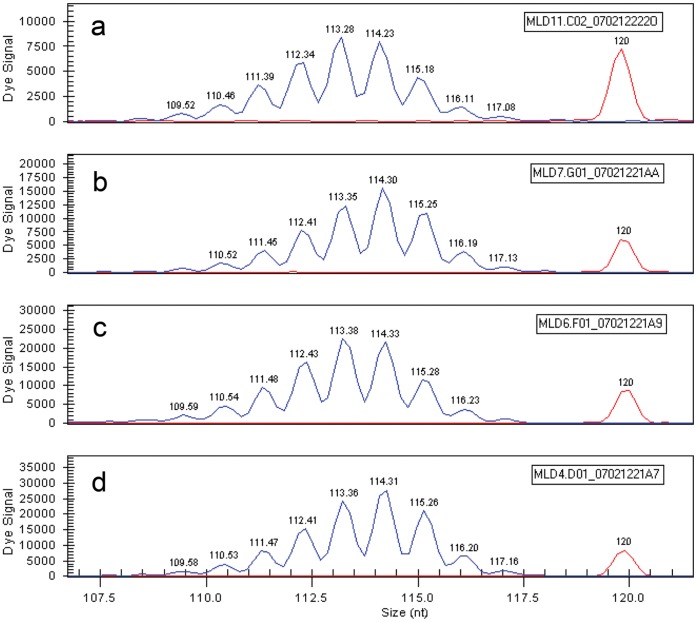
Capillary electrophoresis of PCR products containing the polyA tract. PCR amplicon spanning the *RPGRIP1* polyA insertion was sized by capillary gel electrophoresis. The common electropherogram peak pattern from *RPGRIP1* ^−/−^ MLHDs is represented by dogs MLD7 (b: late-onset affected, 9 y) and MLD4 (d: mid-onset affected, 5 y). In dogs MLD11 (a: clinically normal, 5 y) and MLD6 (c: clinically normal, 9 y), the highest peak in each electropherogram was shifted by 1 bp, to ‘113’, compared to the common PCR fragment peak pattern ‘114’. Note that the majority of the *RPGRIP1* ^−/−^ dogs examined including both clinically affected and normal dogs showed the ‘114’ pattern. Direct cloning and haplotype analysis confirmed MLD6 as heterozygous for polyA_28/29_ while the ‘114’ pattern corresponded to polyA_29/29_.

The mutation associated with *cord1* in a MLHD research colony was a 44 bp insertion (*RPGRIP1* insertion) containing a polyA_29_ tract [8]. Mononucleotide homopolymers are over-represented in many genomes and likely to be unstable during DNA duplication because of slipped strand mispairing, a process that is best characterized in prokaryotes [15], [16], [17], [18]. We hypothesised that the polyA tract of the *RPGRIP1* insertion has size variation resulting in the use of different reading frames downstream of the insertion in different individuals. Initially we attempted to examine the sequence of *RPGRIP1* insertions in MLHDs from the pet population that were *RPGRIP*1^−/−^ by PCR and direct sequencing from both strands. However, using either strand, the sequence read was invariably distorted towards the end of the polynucleotide tract (Figure S1) preventing interpretation. Instead, the PCR amplification products were sized by capillary gel electrophoresis to deduce the size of the polyA tract. For animals from the original colony population of Mellersh et al. [8] a mixture of PCR products were amplified, ranging from 109 bp to 116 bp, with the highest peak at 114 bp (Figure 3b, 3d). Although accurate determination of the length of the polyA run using PCR was problematic, the size range and the position of the highest peak observed by capillary electrophoresis was consistent within individuals when repeated, and consistent variations between different individuals suggested allelic variation of the polyA insert length.

PCR amplification products containing the *RPGRIP1* insertion in *RPGRIP1* ^−/−^ (n = 78) and *RPGRIP1* ^+/−^ (n = 42) MLHDs were sized by capillary electrophoresis. Of the *RPGRIP1* ^−/−^ dogs, 51 were retinal degeneration cases affected at various ages of onset (0.3–15.0 y), one was an acquired retinopathy case (sudden acquired retinal degeneration, SARD, age 6.6 y), three had marginally abnormal fundus with apparently normal visual function (2.4–7.2 y), and 23 had apparently normal vision and fundoscopic appearance (3.8–12.4 y). By comparing the PCR products, we identified a minor second size pattern with the highest peak at 113 bp (Figure 3, compare a, c with b, d) in two apparently clinically normal *RPGRIP1* ^−/−^ dogs (Figure 3a: MLD11, 3.8 y; Figure 3c: MLD6, 7.5 y) that were full-siblings, and an unrelated third dog with apparently normal visual function but a slight fundoscopic abnormality (MLD310, 2.4 y).

#### 2. Absolute sizing by direct cloning from genomic DNA

**Figure 4:**
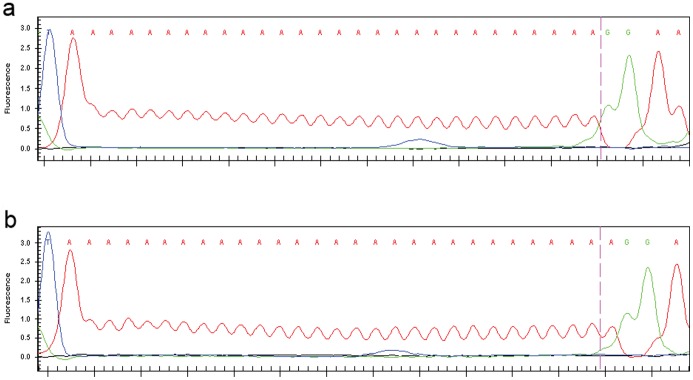
Sequences of the cloned polyA tract of the *RPGRIP1* insertion. Sequences were obtained from genomic DNA fragments cloned and amplified in *E coli*. Shown are electropherograms of single alleles isolated from MLD6 (a, polyA_28_) and from a *cord1*-affected MLHD from the original AHT colony (b: polyA_29_).

To obtain a definite size for the polyA insert, the region was isolated and sequenced without amplification by PCR. Genomic libraries enriched for the region of the *RPGRIP1* insertion were prepared from two *RPGRIP1* ^−/−^ MLHDs with tracts that varied in the electrophoretic size pattern of the PCR product, and clones containing the tract selected for sequencing. After several rounds of pool-screening for clones containing the *RPGRIP1* fragment, a single clone corresponding to one of the two polyA alleles of MLD6 was obtained. When the plasmid was extracted and sequenced, no distortion was seen at the polyA tract. For the MLD6 dog with the minor PCR fragment size pattern, the size of the cloned polyA tract turned out to be 28 bp (Figure 4a), one nucleotide less than the polyA tract cloned from an early-onset *cord1* case in the original MLHD research colony used to identify the mutation [8] (Figure 4b).

#### 3. Haplotype analysis

**Figure 5:**
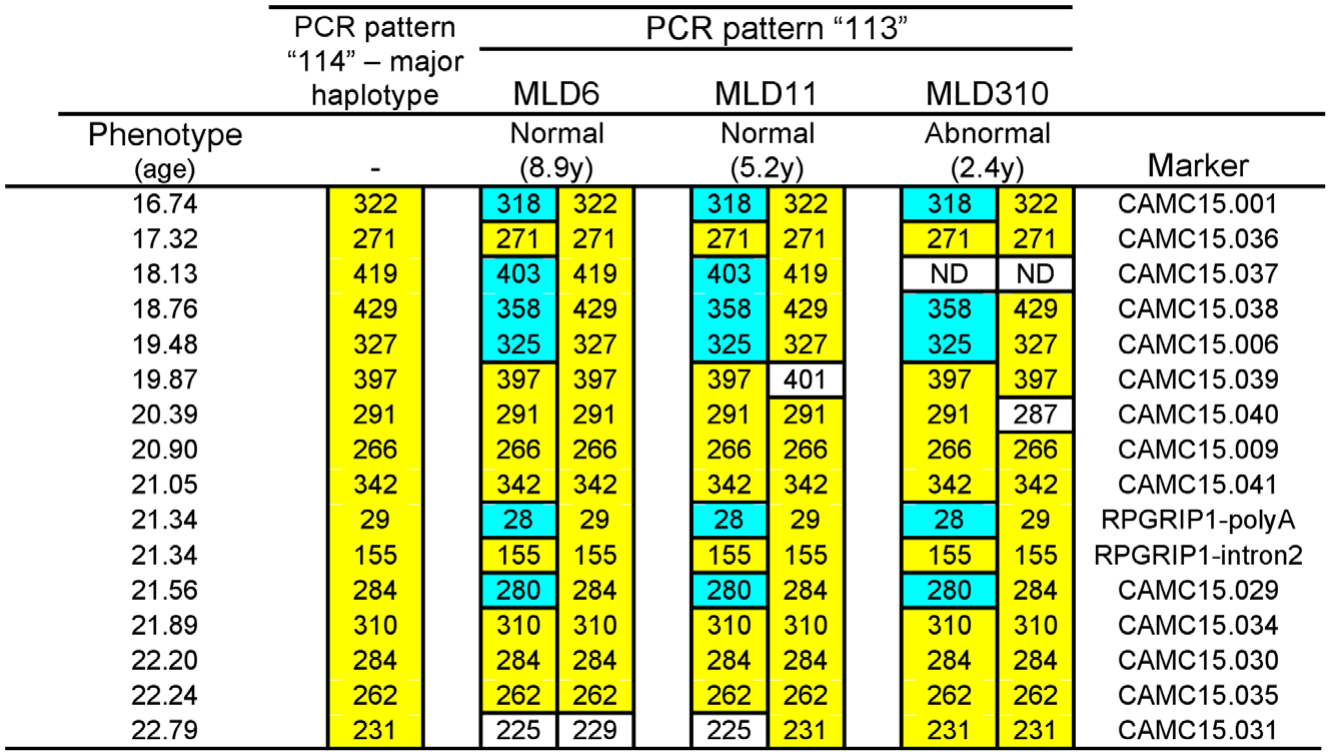
Haplotypes spanning the 6.05 Mb flanking region of *RPGRIP1*. The ‘114’- major haplotype (yellow) corresponds to the haplotype predominant in *RPGRIP1* ^−/−^ dogs, with the common PCR fragment pattern peaking at ‘114’ (A_29_ insert) in Figure 3. Microsatellite marker alleles specific to the dogs with the ‘113’ pattern (A_28_ insert) in Figure 3 are indicated (blue). The polyA_28_ allele was determined by cloning from genomic DNA (*) or PCR-fragment sizing (**).

As the cloning method required a relatively large quantity of DNA from each individual, followed by laborious selection and screenings, haplotypes unique to different polyA tracts were searched for, to identify the different polyA alleles indirectly. Dogs were genotyped using fifteen polymorphic markers previously used for fine-mapping the *cord1* locus [8], and covering a 6.05 Mb region around *RPGRIP1* at intervals of 0.15–0.81 Mb. A unique five position haplotype extending from 16.74 Mb to 21.56 Mb was shared in the three dogs which showed the unique PCR fragment pattern ‘113’ associated with the polyA_28_ insert (Figure 5). Haplotype phasing indicated that all three dogs were heterozygous for the polyA_28_ and polyA_29._ alleles. Among the *RPGRIP1* ^−/−^ dogs that showed the ‘114’ PCR fragment pattern (i.e. homozygous for polyA_29_), extensive phenotypic variations were still observed including early-onset and late-onset retinal degenerations and clinically normal animals.

### Frameshifting Occurs at Long polyA Tracts during Transcription and/or Translation

**Figure 6:**
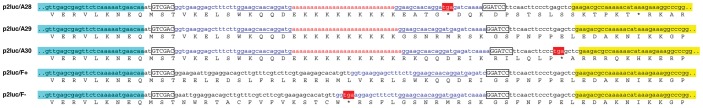
p2 luc constructs used in dual-reporter luciferase assay. DNA sequences and the corresponding amino acids for plasmid constructs with polyA insertions (p2 luc/A_28, 29 and 30_), and in-frame (p2 luc/F+) and *rluc*-only (p2 luc/F-, stop codon upstream of *fluc*). The polyA constructs shown indicate the three possible reading frames after the polyA sequence; only those with (3n-1) adenines, such as A_29_, A_35_, A_38_ and A_41_, lead to an in-frame *fluc*, unless the number of adenines is changed following transcription or the reading frame is altered during translation. (Note that this single base gain in the construct reading frame is specific to this reporter assay. In the cell, A_30_, A_36_, A_39_ and A_42_ would be in frame.) Blue and yellow highlights indicate *Renilla* and firefly gene sequences, respectively. The SalI and BamHI cloning sites are outlined. DNA sequence of the polyA tract is shown in red letters, while the flanking region (exon 3 of *RPGRIP1*) is in blue letters with the 15-bp duplication underlined.

To investigate whether slippage of the transcriptional or translational apparatus on the polyA tract could lead to a protein product despite the presence of a frameshift mutation, a dual-luciferase reporter assay was designed using plasmid constructs with inserts containing polyA tracts of varying sizes from 21 to 43 adenine residues (Figure 6). The tract was bracketed by the 15 bp duplication and inserted in a p2 luc plasmid [19] between two reporter genes, the 5′ *Renilla* luciferase (*rluc*) and the 3′ firefly luciferase (*fluc*).

Following transfection of cultured cells with each construct, the upstream *rluc* would always be expressed, while expression of the downstream *fluc* would depend on the insertion sequence preceding it. These constructs were designed such that only a polyA_3n–1_ tract would place *fluc* in-frame, allowing its expression, but constructs containing a polyA_3n_ or a polyA_3n+1_ tract would introduce a stop codon before *fluc*, preventing its expression unless the reading frame had been restored during transcription and/or translation. Consequently, the ratio of *fluc* expression to *rluc* expression would indicate the occurrence and efficiency of frameshifting during transcription and/or translation. The frequency of frameshifting was established by comparing this ratio to that of a control construct (p2luc/F+) in which both luciferases were in frame, but separated by a random sequence (rather than a polyA tract) of the same insertion size as p2luc/polyA_29_ (Figure 6). The p2luc/polyA and control plasmids were transfected into dog (MDCK) and human (COS-7) cell lines and luciferase expression in cell lysates was measured 24 h post-transfection.

**Figure 7:**
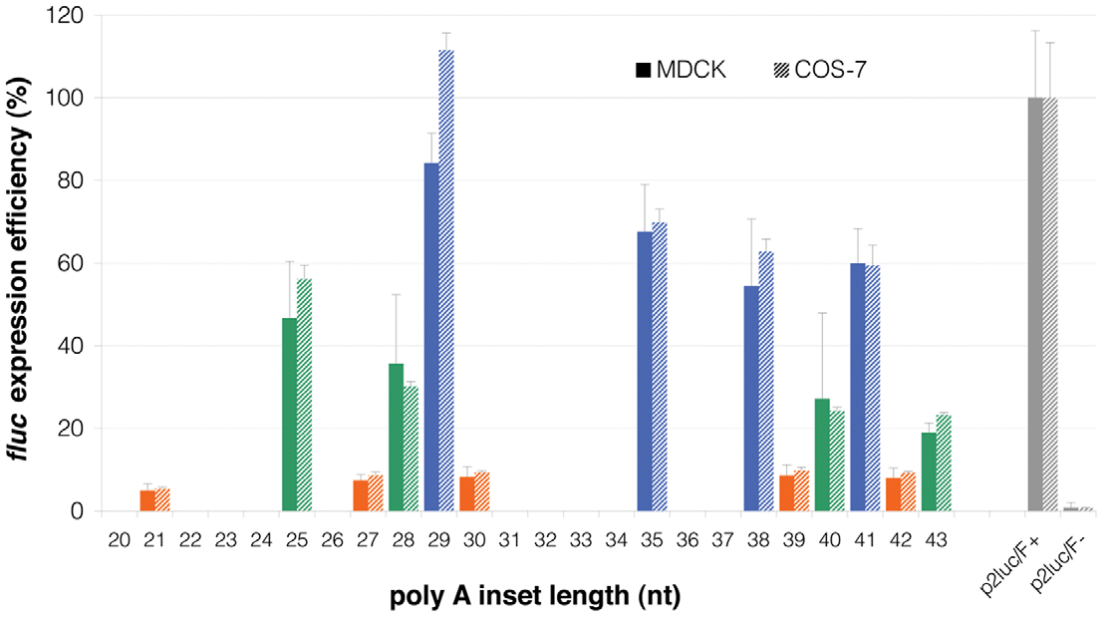
Expression of *fluc* downstream of polyA tracts. The firefly to *Renilla* luciferase expression ratio in p2luc/A_21, 25, 27–30, 35, 38–43_ constructs normalised to the in-frame control (p2luc/F+, 100%) in MDCK cells (solid bars) and COS-7 cells (shaded bars). The green, blue, and orange colours correspond to constructs with polyA_3n+1, 3n−1 and 3n_ inserts, respectively, where in this vector the blue bars represent the constructs that preserve the reading frame of the *fluc* gene. PolyA insert lengths not studied are printed in grey along the X axis. Error bars represent SD of three triplicate assays. p2luc/F- is a negative control for which no *fluc* expression is expected because of a termination codon upstream of the firefly gene.

Surprisingly, all p2luc/polyA constructs, including the putative out-of-frame constructs (polyA_3n_ and polyA_3n+1_), expressed high levels of *fluc* along with *rluc* (Figure 7), indicating the widespread occurrence of frameshifting. In a set of three constructs with successive increasing polyA lengths (polyA_28, 29 and 30_), the in-frame polyA_29_ construct unsurprisingly showed the highest relative level of *fluc* expression. Of the two neighbouring out-of-frame constructs, the polyA_28_ construct showed higher relative *rluc* expression (42.4% c.f. polyA_29_) than the polyA_30_ construct (9.8% c.f. polyA_29_) (Figure 7) indicating that net +1 frameshifting occurs more frequently than net +2 or −1 frameshifting. This observation was in agreement with a related set of constructs (polyA_40, 41 and 42_), although the relative levels of expression in the out-of-frame constructs was even higher in these longer polyA runs (44.0% for the +1 frameshift and 15.0% for the +2/−1 frameshift, compared with the polyA_41_ in-frame construct).

Relative *fluc* expression from the p2luc/polyA_29_ in-frame construct was decreased to 84% of that measured with p2luc/F+ positive control in MDCK cells (Figure 7), consistent with frameshifting also occurring on templates that maintain reading frame when transcribed correctly, and leading to some transcripts being non-functional for *fluc* expression. However, this was not a statistically significant difference, nor was it seen in COS cells. Nevertheless, it is clear that high-levels of frameshifting occur with the out-of-frame insertions. Luciferase assays in COS-7 cells resulted in very comparable observations with those of MDCK cells indicating that these phenomena play a role across species.

## Discussion

In *cord1*, an insertion causing a frameshift in a gene that is believed to be absolutely required for normal photoreceptor function has a variable and sometimes subclinical effect on vision. In this paper, we have examined three possible explanations for this. We have examined the transcript structures of the gene. In common with a recent study from Kuznetsova et al. [12], our data shows that canine *RPGRIP1* has a very complex splicing pattern. An additional upstream non-coding exon has caused us to renumber all exons, so that the exon containing the insertion becomes exon 3. In addition, 5′ RACE suggests the presence of two internal promoters (albeit weak ones as indicated by expression levels in qRT-PCR). In addition to variations described by Kuznetsova et al. [12], we have shown further alternate splicing at the 5′ end of the gene. For all but two of these canine *RPGRIP1* isoforms (CR1 and CR9 in Figure 1) orthologous isoforms have been previously isolated as either bovine, mouse or macaque full length *RPGRIP1* cDNA clones or EST [10], [11], [20] and Genbank entries (see Table S2).

We have not attempted to verify the presence of differentially spliced isoforms by northern blotting as the technique is relatively imprecise and of modest sensitivity, and has not been able to define such isoforms in previous studies of *RPGRIP1* expression [11], [20], [21]. Instead qRT-PCR was used to quantitate RNA’s from a variety of exon pairs. The highest mRNA concentration amongst the *RPGRIP1* regions examined was close to the 5′ end of the transcript at exon 2/3. RNA concentration from those products with downstream exons examined here was an order of magnitude lower than that of exon 2/3 except for an exon-skipping product exon 14b/18 whose expression level was ∼1% of exon 2/3. There was little difference between either the proportion or the concentration of different splice variants between *RPGRIP1* ^−L/−L^ and *RPGRIP1* ^+/+^. Both this study and that of Kuznetsova et al. [12] note transcripts where a set of exons following exons 3 and 14 (a and b) are omitted. One transcript (CR9, Figure 1c) encodes an open reading frame whilst skipping the insertion site within exon 3. However this transcript, which has not been reported by others, proved too rare to be measurable by qRT-PCR and if translated will give only small amounts of a grossly shorter peptide unlikely to be able to substitute for all functions of the full length protein. In particular it would lack a conserved coiled coil protein interaction domain (of the SMC family) and two protein kinase C like domains (C2) of the full length protein (see Figure 1), although retaining a nuclear domain and an RPGR binding domain [11], [12], [22], [23]. In short, these data give no indication that any large proportion of *RPGRIP1* ^−/−^ mRNA retains full functionality after differential splicing to an isoform not containing the mutated exon. Even if some functions may be retained in CR9 isoform transcripts, only very small quantities of spliced RNAs escape the mutation this way.

Simple tandem repeats and polynucleotide tracts are both well known to be less stable in replication than more complex sequences [24], [25]. We have previously shown that the length of the polyA insert in *RPGRIP1* varies between breeds [9]. Here we show that there is also length variation in the tract between different individuals of the same breed (MLHD). The most common mutant allele in our study population has 29 adenine residues, but a second allele has only 28 adenines. The effect of this change on the severity of the mutation should be slight. Like the polyA_29_ insert, the polyA_28_ variant is also out-of-frame compared with the wild-type, and in this case, truncation of the protein is expected to occur only five amino acids downstream from the poly-Lysine run encoded by the polyA insert, within exon 3, rather than in exon 4, as is the case for polyA_29_ inserts. Based on differential *fluc* expression efficiency in the dual-reporter assay, a polyA_29_ allele (equivalent to the p2luc/A_28_ construct) could result in higher levels of in-frame transcripts compared to polyA_28_ (equivalent to p2luc/A_27_). But lack of correlation between the polyA genotype (polyA_28/29_ or polyA_29/29_) and disease severity suggests that in practice such variations are insufficient to have marked effects on phenotype of heterozygotes.

The polyA_28_ insertion variant is associated with a distinct haplotype on both sides of the insertion site. A 280 bp allele at position 21.56 Mb has also been seen in at least one animal with polyA_29_ in the insert, and this microsatellite appears hypermutable, as in addition to the predominant 284 bp allele three other alleles have been found at this position in polyA_29_ dogs [9]. The remainder of the haplotype associated with these three polyA_28_ chromosomes extends across 4.5 Mb. Two of these three dogs are siblings, but the extended haplotype in the third dog, also suggests a recent common ancestor in which the polyA_28_ allele arose. This haplotype allows a rapid and accurate test for the presence of this allele where genotyping of the insertion itself presents difficulties. We have not yet found any individuals in any breed that have an in-frame variant polyA tract (for example, polyA_27_ or polyA_30_). These individuals may exist, and may have avoided clinical attention because they show no disease. We have already shown that the polyA_29_ allele can be associated with both early and late onset disease and have no evidence that polyA_28_ dogs show a different pattern [9].

It is notable that there is little or no reduction in the level of the *RPGRIP1* transcripts in the retina of beagles that have the insertion mutation compared with those that do not, suggesting there is little nonsense-mediated decay of these transcripts. This result would be expected if *RPGRIP1* transcripts remain translationally active even when they contain the insertion.

The dual-luciferase reporter assay gave a more rigorous test of whether the *RPGRIP1* insertion site mutation caused complete loss of expression of downstream protein coding sequences. In this assay, we found a general tendency for the level of expression downstream of the insertion to get lower as the polyA tract insertion length increases. More remarkably, the results showed that out-of-frame polyA tracts permitted a very high level of access to the downstream reporter gene in either canine or primate kidney cell lines. In fact, the level of +1 frameshifting exceeds 40% for polyA tracts of 28 or 40 bases in length. This frameshifting may account for the lack of major transcript loss by nonsense-mediated decay in *RPGRIP1* ^−/−^ dogs. There is also substantial −1 (or +2) frameshifting, so that there is easily measurable protein expression downstream of these constructs. Such high rates of frameshifting caused by polymerase slippage on polyA tracts within genes have been observed in endosymbiont bacteria [26]. Substantial levels of transcriptional frameshifting on polyA tracts have also been measured directly in *E. coli* [17] as well as in *S. flexneri* where transcriptional slippage at polyA_9_ and polyA_10_ tracts plays an active role in controlling production of secretion apparatus components [27]. High levels of transcription errors have been reported from human *TGFBR2* and *ATRX* genes containing polyA_10_ and polyT_13_ runs, but these analyses used a PCR step [28]. Yeast RNA Pol II is known to slip frequently on artificial templates consisting of polydA^+^ tracts greater than 11 bp, with a bias towards adding a nucleotide to the transcript [29]. In mammalian COS-7 cells, reading frame restoration efficiency of ∼10% at a polyA_8_ tract of the apoB mutant has been attributed to transcriptional insertion of an extra adenine [30]. These are in accordance with our polyA_21–43_ reporter assay where net +1 frameshifting outweighed that of net −1. Due to the nature of this reporter assay, transcription and translation phases could not be assessed independently. Therefore, the origin of the reading frame restoration remains to be determined. Indeed, expression of DNA polymerase III subunits γ and τ is achieved by contrasting mechanisms in *T. thermophilus* and *E. coli*; polyT_9_ transcriptional slippage in the former, and translational frameshifting in the latter [31]. Similarly in canine cyclic neutropenia an polyA_9_ tract is mutated to polyA_10_ in affected animals, but there is partial rescue of homozygotes by slippage, and both addition and loss of A residues is seen for about 10% of transcripts in each case [32]. We believe that the levels of frameshifting reported here are unprecedented in eukaryotic cellular genes, and compare favourably with documented sites of high-level programmed ribosomal frameshifting in virus gene expression [33].

The frameshifting observed here can contribute to leakiness of the *RPGRIP1* ^−/−^ mutation *in vivo* in *cord1* dogs, accounting for the survival of vision in some affected animals until late in life [9]. We have recently identified a second locus as a modifier which, when homozygous in *RPGRIP1* ^−/−^ dogs, causes earlier onset of the disease [34]. All individuals from the original research colony in which *RPGRIP1* ^−/−^ was identified [8] were shown to be homozygous for the early onset modifier. It is possible that leakiness at the *RPGRIP1* ^−/−^ mutation is sufficient to prevent severe disease except in the presence of the homozygous mutation of the modifier causing early onset. We are now exploring the nature of the interaction between these two loci.

## Methods

### Ethics Statement

Retinal tissues were obtained from *RPGRIP1* ^+/+^ and *RPGRIP1* ^−L/−L^ clinically normal beagle dogs when put down for other experimental purposes at the VMC, University of Tokyo, after ethical review and consent from the Animal Care and Use Committee of the Faculty of Agriculture, University of Tokyo, whose guidelines are developed under the “Law for the Humane Treatment and Management of Animals”, 2000. (Permission held by Prof N. Sasaki.) Tissues were salvaged from dogs *post mortem*, and *RPGRIP1* mutant status was not a factor in deciding which dogs were euthanized, but was an adventitious finding when blood specimens from the dogs were examined during a canine population survey for the mutation [9]. All DNAs used in this work were from archival blood and buccal samples as previously described [8], [29], [34].

### Canine DNA and Retinal RNA

Archived DNA, obtained from blood and buccal samples from pet or colony dogs of known ophthalmoscopic and visual status, has been described previously [8], [9], [34]. Retinal tissues were obtained from *RPGRIP1* ^+/+^ and *RPGRIP1* ^−L/−L^ clinically normal beagle dogs. Neuroretina specimens were stored at −20°C in RNAlater (Qiagen) until RNA could be prepared using the RNeasy mini-kit (Qiagen) according to the manufacturer’s protocol.

### cDNA Synthesis, RACE and RT-PCR

Primer pairs were designed to amplify the entire predicted canine coding sequence with overlapping PCR fragments (Table S3). Reverse transcription-PCR used 4 µg total retinal RNA samples and standard techniques. Products were run on agarose gels, purified by excision and sequenced. 5′ Rapid Amplification of cDNA Ends (5′RACE) used a commercial kit (SMARTer RACE cDNA Amplification Kit, Clontech Laboratories, Inc.) and 1–5 ug samples of total retinal RNA. A variety of *RPGRIP1* specific genomic primers were used to attempt to find all internal transcription start sites. RACE and RT-PCR products were sequenced by Sanger sequencing (3730×l DNA Analyser, ABI) and sequences were deposited in GenBank (Accession# KC107780-KC107785).

### Quantitative Real Time-PCR

qRT-PCR used a Rotorgene 3000 (Rotorgene) and Maxima® SYBR Green qPCR Master Mix (2X) (Fermentas) according to the manufacturer’s instructions. Primer sets encompassing exon boundaries of the various canine *RPGRIP1* transcripts were designed from the transcripts identified in Figure 1 (Tables S2, S3), including a primer set amplifying the alternatively spliced transcript containing exons 14b/18. After optimisation of each primer set to ensure very high cycling efficiency (>1.9 fold amplification per cycle), complete specificity, cDNA dependency and lack of reaction on genomic DNA, qRT-PCR reactions were carried out individually for each primer set. For each test reaction, equal amounts of retinal cDNA samples were quantitated by spectrophotometry for use as templates. Plasmid clones of the appropriate RT-PCR product in the vector pCR®2.1-TOPO (Invitrogen) were used as copy number standards for each primer pair so that original numbers of cDNA template molecules specific to each primer pair could be calculated and compared across reactions. Test reactions were performed in triplicate and repeated in duplicate experiments.

### Sizing the *RPGRIP1* Insertion from Genomic DNA

Because genomic PCR and sequencing failed to give a definite size for the polyA insertion, a route not requiring PCR amplification, (cloning with pool selection) was used to derive the insertion sequence from two dogs showing distinct PCR capillary electrophoresis patterns on insert sizing.

EcoRI and BamHI digestion of canine genomic DNA produces a 1,935 bp fragment containing the *RPGRIP1* insertion site. To enrich for DNA fragments containing this sequence, this digestion product was further cut to completion with AciI, HhaI and TaqI which have no target sites in the 1935 bp fragment. DNA of 1,935±300 bp was collected following agarose gel electrophoresis of the digestion products, and ligated into pUC19. After electroporation into *E. coli* (ElectroMAX™ DH5α-E™ Cells, Invitrogen), *RPGRIP1* DNA containing colonies were isolated from libraries for each individual by using a replica plating and dilution cycle, and a PCR assay for the presence of *RPGRIP1* sequence within plasmid-containing colonies in the pool of colonies plated. *RPGRIP1* positive plasmids were re-isolated and sequenced without using PCR.

For haplotype reconstruction, microsatellite markers were typed as previously described [9] using custom designed primers [9] (Table S4) and capillary gel electrophoresis.

### Reporter Constructs and Dual-luciferase Assay

Inserts containing variable length of polyA_n_ (A_21_, A_25_, each of A_27_–A_30_, A_35_ and each of A_38_–A_43_) with 55 bp of the surrounding *RPGRIP1* sequence were obtained by PCR amplification of the *RPGRIP1* insertion region in a MLHD (polyA_29_, *RPGRIP1* ^−/−^) and a beagle (polyA_∼44_, *RPGRIP1* ^−L/−L^). Amplification products (Table S5) were inserted in a p2luc plasmid [19] between two reporter genes, an upstream *Renilla* luciferase (*rluc*) and a downstream firefly luciferase (*fluc)*. In these constructs, the downstream reading frame is in frame when the number of bases, n, in the polyA_n_ tract, is such that n+1 is divisible by three (Figure 6). In addition, an in-frame positive control (p2luc/F+) and a negative control with a stop codon upstream of the mutation insertion site (p2luc/F-) were constructed. Each control plasmid harboured an insert of a complex sequence from *RPGRIP1* equivalent in size to that of the p2luc/A_29_ construct. Constructs were cloned and polyA tract lengths confirmed by sequencing. For luminescence experiments, cloned reporter plasmids were transfected into sub-confluent MDCK and COS-7 cells using the liposome method and a FuGene®6 (Roche) reagent, and incubated at 37°C for 48 h before assaying lysates of the washed cells. Dual-luciferase reporter assays were performed using Luciferase Assay Reagent II (Promega), followed by Stop & Glo® Reagent (Promega) according to the manufacturer’s protocols. Assays were performed in triplicate in all experiments, with three independent experiments for MDCK and one experiment for COS-7.

## Supporting Information

Figure S1 **Direct sequencing of PCR products from** ***RPGRIP1*** **exon 3 DNA spanning the polyA tract from a single** ***RPGRIP1*** **^−/−^ dog.** The electropherogram signal is distorted through the presence in the PCR of multiple products with different numbers of A residues in the amplified homopolymer, showing the difficulty of using PCR to analyse this polyA insertion.
(TIF)

Table S1 **Exonic polymorphisms across the minimal conserved haplotype around the** ***RPGRIP1*** **gene in an insert homozygous** ***cord1*** **dog.** The region of canine autosome 15 from 20,218,076-21,962,566 (CanFam 2.0 assembly) was captured using DNA capture with the SureSelect Target Enrichment system (Agilent) and sequenced using Illumina GA paired-end (120 bp reads) technology for two *RPGRIP1* ^−/−^ early-onset and four *RPGRIP1* ^−/−^ late-onset *cord1* MLHDs. Captured SNP changes from CanFam 2.0 in one of the early-onset dogs are shown for *RPGRIP1* and for roughly 200 kb on either side of the gene.
(DOC)

Table S2 **Transcripts in Genbank with equivalent splice patterns to those reported here.**
(DOC)

Table S3 **Primers used in cDNA analysis and for qRT-PCR.**
(DOC)

Table S4 **Primers used for microsatellite genotyping.** These primers were used in checking haplotypes around *RPGRIP1*.
(DOC)

Table S5 **Primers used for analyzing the polyA tract and for constructing plasmids for dual-reporter assay.**
(DOC)
