# Supplementary figures and images for: Multiple Mechanisms Contribute to Leakiness of a Frameshift Mutation in Canine Cone-Rod Dystrophy
Source: PLoS One. 2012 Dec 12;7(12):e51598. doi: 10.1371/journal.pone.0051598 (PMC3520932; doi:10.1371/journal.pone.0051598)

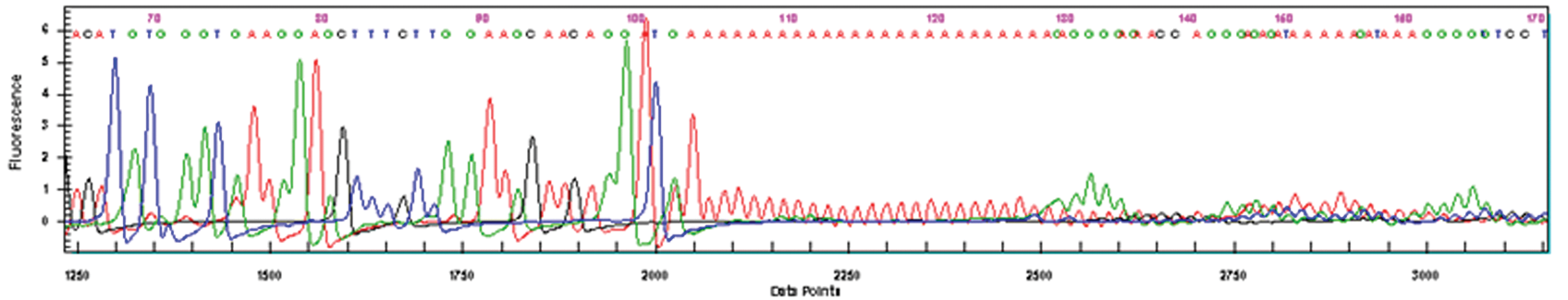

Supplement: Figure S1 — Direct sequencing of PCR products from RPGRIP1 exon 3 DNA spanning the polyA tract from a single RPGRIP1 −/− dog. The electropherogram signal is distorted through the presence in the PCR of multiple products with different numbers of A residues in the amplified homopolymer, showing the difficulty of using PCR to analyse this polyA insertion. (TIF) [file pone.0051598.s001.tif]
