# Supplementary material for: Multiple Mechanisms Contribute to Leakiness of a Frameshift Mutation in Canine Cone-Rod Dystrophy
Source: PLoS One. 2012 Dec 12;7(12):e51598. doi: 10.1371/journal.pone.0051598 (PMC3520932; doi:10.1371/journal.pone.0051598)
Supplement: Table S1 — Exonic polymorphisms across the minimal conserved haplotype around the RPGRIP1 gene in an insert homozygous cord1 dog. The region of canine autosome 15 from 20,218,076-21,962,566 (CanFam 2.0 assembly) was captured using DNA capture with the SureSelect Target Enrichment system (Agilent) and sequenced using Illumina GA paired-end (120 bp reads) technology for two RPGRIP1 −/− early-onset and four RPGRIP1 −/− late-onset cord1 MLHDs. Captured SNP changes from CanFam 2.0 in one of the early-onset dogs are shown for RPGRIP1 and for roughly 200 kb on either side of the gene. (DOC) [file pone.0051598.s002.doc]

## Table S1

| ## ENSEMBL VARIANT EFFECT PREDICTOR v2.0 | | |  |  |  |  |  |  |  | |  |
| --- | --- | --- | --- | --- | --- | --- | --- | --- | --- | --- | --- |
| #Uploaded_variation | Allele | Gene | Consequence | mRNA posn | CDS posn | Protein posn | Amino_acids | Codons | Existing variation | | Notes (for those genes with exonic polymorphisms) |
| 15_21097201_C/T | T | METLL17 methyltransferase like 17 | UPSTREAM |  |  |  |  |  | - | |  |
| 15_21099230_C/T | T | METLL17 methyltransferase like 17 | UPSTREAM |  |  |  |  |  | - | |  |
| 15_21099263_G/C | C | METLL17 | UPSTREAM |  |  |  |  |  | - | |  |
| 15_21103289_G/A | A | SLC39A2 solute carrier 39A2 (Zn transporter) | UPSTREAM |  |  |  |  |  | - | |  |
| 15_21105116_A/G | G | METLL17 | INTRONIC |  |  |  |  |  | - | |  |
| 15_21106546_A/G | G | METLL17 | DOWNSTREAM |  |  |  |  |  | - | |  |
| 15_21107960_C/T | T | SLC39A2 | INTRONIC |  |  |  |  |  | - | |  |
| 15_21111087_G/A | A | NDRG2 N-myc downregulated gene family member 2 | DOWNSTREAM |  |  |  |  |  | - | |  |
| 15_21111195_G/A | A | SLC39A2 | DOWNSTREAM |  |  |  |  |  | rs22420607 | | |
| 15_21111917_T/C | C | SLC39A2 | DOWNSTREAM |  |  |  |  |  | - |  | |
| 15_21112215_A/G | G | NDRG2 | DOWNSTREAM |  |  |  |  |  | - |  | |
| 15_21114148_G/T | T | NDRG2 | DOWNSTREAM |  |  |  |  |  | - |  | |
| 15_21115970_A/C | C | NDRG2 | INTRONIC |  |  |  |  |  | - |  | |
| 15_21119535_A/G | G | NDRG2 | INTRONIC |  |  |  |  |  | rs22364442 | | |
| 15_21119688_C/T | T | NDRG2 | INTRONIC |  |  |  |  |  | rs22364443 | | |
| 15_21120019_G/A | A | NDRG2 | INTRONIC |  |  |  |  |  | - |  | |
| 15_21120237_A/G | G | NDRG2 | INTRONIC |  |  |  |  |  | rs22364444 | | |
| 15_21123093_T/A | A | TPPP2 tubulin polymerisation-promoting protein 2 | UPSTREAM |  |  |  |  |  | - | TPPP2 fn unknown. May bind tubulin but has no microtubule bundling activity. Not expressed in eye. | |
| 15_21123155_G/C | C | TPPP2 | UPSTREAM |  |  |  |  |  | - |  | |
| 15_21123164_G/C | C | TPPP2 | UPSTREAM |  |  |  |  |  | - |  | |
| 15_21123166_A/C | C | NDRG2 | UPSTREAM |  |  |  |  |  | - |  | |
| 15_21123197_A/C | C | NDRG2 | UPSTREAM |  |  |  |  |  | - |  | |
| 15_21123204_A/C | C | TPPP2 | UPSTREAM |  |  |  |  |  | - |  | |
| 15_21123218_A/C | C | NDRG2 | UPSTREAM |  |  |  |  |  | - |  | |
| 15_21123225_T/A | A | TPPP2 | UPSTREAM |  |  |  |  |  | - |  | |
| 15_21123238_A/C | C | TPPP2 | UPSTREAM |  |  |  |  |  | - |  | |
| 15_21123313_T/C | C | TPPP2 | UPSTREAM |  |  |  |  |  | - |  | |
| 15_21123338_T/C | C | NDRG2 | UPSTREAM |  |  |  |  |  | - |  | |
| 15_21123348_T/C | C | NDRG2 | UPSTREAM |  |  |  |  |  | - |  | |
| 15_21123879_A/G | G | NDRG2 | UPSTREAM |  |  |  |  |  | - |  | |
| 15_21124417_A/G | G | NDRG2 | UPSTREAM |  |  |  |  |  | rs22420608 | | |
| 15_21124725_G/A | A | NDRG2 | UPSTREAM |  |  |  |  |  | - |  | |
| 15_21125455_G/C | C | RNASE13 | DOWNSTREAM |  |  |  |  |  | - |  | |
| 15_21126195_A/C | C | TPPP2 | UPSTREAM |  |  |  |  |  | - |  | |
| 15_21126640_A/G | G | RNASE13 | DOWNSTREAM |  |  |  |  |  | - |  | |
| 15_21126661_A/G | G | TPPP2 | UPSTREAM |  |  |  |  |  | - |  | |
| 15_21126671_T/G | G | RNASE13 | DOWNSTREAM |  |  |  |  |  | - |  | |
| 15_21126727_T/G | G | TPPP2 | UPSTREAM |  |  |  |  |  | - |  | |
| 15_21126770_G/A | A | RNASE13 | DOWNSTREAM |  |  |  |  |  | - |  | |
| 15_21126826_G/T | T | TPPP2 | UPSTREAM |  |  |  |  |  | - |  | |
| 15_21126913_T/A | A | TPPP2 | NON_SYNONYMOUS_CODING | 61 | 7 | 3 | S/T | Tca/Aca | - |  | |
| 15_21126914_C/A | A | TPPP2 | STOP_GAINED | 62 | 8 | 3 | S/* | tCa/tAa | - |  | |
| 15_21127015_A/T | T | TPPP2 | NON_SYNONYMOUS_CODING | 163 | 109 | 37 | S/C | Agt/Tgt | - |  | |
| 15_21128351_C/A | A | TPPP2 | STOP_GAINED | 540 | 486 | 162 | Y/* | taC/taA | - |  | |
| 15_21128843_T/G | G | RNASE13 | DOWNSTREAM |  |  |  |  |  | - |  | |
| 15_21128858_C/T | T | TPPP2 | DOWNSTREAM |  |  |  |  |  | - |  | |
| 15_21129458_A/G | G | RNASE13 | DOWNSTREAM |  |  |  |  |  | rs22419271 | | |
| 15_21129913_A/G | G | RNASE13 | DOWNSTREAM |  |  |  |  |  | - |  | |
| 15_21130923_T/C | C | TPPP2 | DOWNSTREAM |  |  |  |  |  | rs22420612 | | |
| 15_21131097_A/G | G | RNASE13 | UPSTREAM |  |  |  |  |  | - |  | |
| 15_21132798_A/G | G | TPPP2 | DOWNSTREAM |  |  |  |  |  | rs22420622 | | |
| 15_21133083_A/C | C | RNASE13 | UPSTREAM |  |  |  |  |  | - |  | |
| 15_21134203_G/A | A | RNASE13 | UPSTREAM |  |  |  |  |  | - |  | |
| 15_21135492_A/G | G | RNASE13 | UPSTREAM |  |  |  |  |  | rs22422588 | | |
| 15_21164049_G/C | C | ARHGEF40 Rho guanine nucleotide exchange factor 40 | UPSTREAM |  |  |  |  |  | - | Activate low mwt GTPases at plasma membrane | |
| 15_21165114_C/T | T | ARHGEF40 | UPSTREAM |  |  |  |  |  | - |  | |
| 15_21171856_G/A | A | ARHGEF40 | DOWNSTREAM |  |  |  |  |  | - |  | |
| 15_21175525_G/A | A | ARHGEF40 | UPSTREAM |  |  |  |  |  | - |  | |
| 15_21178761_C/G | G | ARHGEF40 | SYNONYMOUS_CODING | 1752 | 1752 | 584 | A | gcC/gcG | - |  | |
| 15_21178909_A/G | G | ARHGEF40 | NON_SYNONYMOUS_CODING | 1900 | 1900 | 634 | I/V | Atc/Gtc | - |  | |
| 15_21179028_C/G | G | ARHGEF40 | SPLICE_SITE,SYNONYMOUS_CODING | 2019 | 2019 | 673 | A | gcC/gcG | - |  | |
| 15_21179034_T/G | G | ARHGEF40 | SPLICE_SITE,INTRONIC | |  |  |  |  | - |  | |
| 15_21179855_C/A | A | ARHGEF40 | NON_SYNONYMOUS_CODING | 2043 | 2043 | 681 | D/E | gaC/gaA | - |  | |
| 15_21179855_C/A | A | ARHGEF40 | NON_SYNONYMOUS_CODING | 24 | 24 | 8 | D/E | gaC/gaA | - |  | |
| 15_21180977_T/A | A | ARHGEF40 | INTRONIC |  |  |  |  |  | - |  | |
| 15_21181695_C/G | G | ZNF219 Zinc Finger Protein 219 | DOWNSTREAM |  |  |  |  |  | - |  | |
| 15_21181707_A/G | G | ARHGEF40 | INTRONIC |  |  |  |  |  | - |  | |
| 15_21181709_A/G | G | ZNF219 | DOWNSTREAM |  |  |  |  |  | - |  | |
| 15_21181720_A/G | G | ARHGEF40 | INTRONIC |  |  |  |  |  | - |  | |
| 15_21181727_C/A | A | ZNF219 | DOWNSTREAM |  |  |  |  |  | - |  | |
| 15_21181730_A/G | G | ARHGEF40 | INTRONIC |  |  |  |  |  | - |  | |
| 15_21181747_C/G | G | ARHGEF40 | INTRONIC |  |  |  |  |  | - |  | |
| 15_21181759_A/G | G | ZNF219 | DOWNSTREAM |  |  |  |  |  | - |  | |
| 15_21181769_A/G | G | ARHGEF40 | INTRONIC |  |  |  |  |  | - |  | |
| 15_21181781_A/G | G | ZNF219 | DOWNSTREAM |  |  |  |  |  | - |  | |
| 15_21181789_C/G | G | ARHGEF40 | INTRONIC |  |  |  |  |  | - |  | |
| 15_21184151_G/T | T | ZNF219 | DOWNSTREAM |  |  |  |  |  | - |  | |
| 15_21184355_T/G | G | ARHGEF40 | DOWNSTREAM |  |  |  |  |  | - |  | |
| 15_21184787_G/A | A | ZNF219 | DOWNSTREAM |  |  |  |  |  | - |  | |
| 15_21191991_T/G | G | ZNF219 | UPSTREAM |  |  |  |  |  | - |  | |
| 15_21193436_T/G | G | C14ORF176 chromosome 14 open reading frame 176 | UPSTREAM |  |  |  |  |  | - |  | |
| 15_21193437_T/C | C | ZNF219 | UPSTREAM |  |  |  |  |  | - |  | |
| 15_21194489_T/G | G | C14ORF176 | UPSTREAM |  |  |  |  |  | - |  | |
| 15_21194490_T/A | A | C14ORF176 | UPSTREAM |  |  |  |  |  | - |  | |
| 15_21194787_T/C | C | C14ORF176 | UPSTREAM |  |  |  |  |  | - |  | |
| 15_21195312_A/G | G | C14ORF176 | UPSTREAM |  |  |  |  |  | - |  | |
| 15_21195566_A/G | G | C14ORF176 | UPSTREAM |  |  |  |  |  | rs22434226 | | |
| 15_21195580_A/T | T | C14ORF176 | UPSTREAM |  |  |  |  |  | - |  | |
| 15_21197363_A/G | G | C14ORF176 | INTRONIC |  |  |  |  |  | - |  | |
| 15_21198254_G/A | A | C14ORF176 | INTRONIC |  |  |  |  |  | - |  | |
| 15_21199933_A/G | G | C14ORF176 | DOWNSTREAM |  |  |  |  |  | rs22423757 | | |
| 15_21200891_T/C | C | C14ORF176 | DOWNSTREAM |  |  |  |  |  | rs22434227 | | |
| 15_21202395_A/G | G | C14ORF176 | DOWNSTREAM |  |  |  |  |  | rs9104640 |  | |
| 15_21202933_A/G | G | C14ORF176 | DOWNSTREAM |  |  |  |  |  | - |  | |
| 15_21252455_T/A | A | HNRNPCL1 heterogeneous nuclear ribonucleoprotein C-like 1 | INTRONIC |  |  |  |  |  | rs22434256 | | |
| 15_21258339_T/C | C | HNRNPCL1 | INTRONIC |  |  |  |  |  | - |  | |
| 15_21258343_T/C | C | HNRNPCL1 | INTRONIC |  |  |  |  |  | - |  | |
| 15_21262602_A/T | T | HNRNPCL1 | INTRONIC |  |  |  |  |  | - |  | |
| 15_21264132_C/A | A | HNRNPCL1 | INTRONIC |  |  |  |  |  | - |  | |
| 15_21264138_G/A | A | HNRNPCL1 | INTRONIC |  |  |  |  |  | - |  | |
| 15_21264141_C/A | A | HNRNPCL1 | INTRONIC |  |  |  |  |  | - |  | |
| 15_21338012_T/A | A | RPGRIP1 | UPSTREAM |  |  |  |  |  | rs22406120 | Primary mutation in Cord1 is insertion in exon3 | |
| 15_21338013_T/A | A | RPGRIP1 | UPSTREAM |  |  |  |  |  | rs22406122 | | |
| 15_21338816_G/A | A | RPGRIP1 | SYNONYMOUS_CODING | 135 | 135 | 45 | E | gaG/gaA | - |  | |
| 15_21343075_G/A | A | RPGRIP1 | INTRONIC |  |  |  |  |  | - |  | |
| 15_21343386_C/T | T | RPGRIP1 | INTRONIC |  |  |  |  |  | rs22406123 | | |
| 15_21344030_T/A | A | RPGRIP1 | INTRONIC |  |  |  |  |  | - |  | |
| 15_21344135_T/C | C | RPGRIP1 | INTRONIC |  |  |  |  |  | - |  | |
| 15_21344136_T/G | G | RPGRIP1 | INTRONIC |  |  |  |  |  | - |  | |
| 15_21344141_G/C | C | RPGRIP1 | INTRONIC |  |  |  |  |  | - |  | |
| 15_21344370_C/T | T | RPGRIP1 | INTRONIC |  |  |  |  |  | - |  | |
| 15_21345071_T/C | C | RPGRIP1 | INTRONIC |  |  |  |  |  | - |  | |
| 15_21345304_T/C | C | RPGRIP1 | INTRONIC |  |  |  |  |  | - |  | |
| 15_21345367_C/G | G | RPGRIP1 | INTRONIC |  |  |  |  |  | - |  | |
| 15_21345499_C/G | G | RPGRIP1 | INTRONIC |  |  |  |  |  | rs22406131 | | |
| 15_21346561_G/C | C | RPGRIP1 | INTRONIC |  |  |  |  |  | rs22406133 | | |
| 15_21346690_T/A | A | RPGRIP1 | INTRONIC |  |  |  |  |  | rs22406135 | | |
| 15_21346801_A/G | G | RPGRIP1 | INTRONIC |  |  |  |  |  | rs22406158 | | |
| 15_21346964_T/G | G | RPGRIP1 | NON_SYNONYMOUS_CODING | 645 | 645 | 215 | D/E | gaT/gaG | - |  | |
| 15_21348008_A/T | T | RPGRIP1 | INTRONIC |  |  |  |  |  | rs22406160 | | |
| 15_21348629_T/C | C | RPGRIP1 | INTRONIC |  |  |  |  |  | rs22406163 | | |
| 15_21348648_A/G | G | RPGRIP1 | INTRONIC |  |  |  |  |  | rs22406164 | | |
| 15_21349112_G/A | A | RPGRIP1 | INTRONIC |  |  |  |  |  | rs22406167 | | |
| 15_21349426_T/C | C | RPGRIP1 | INTRONIC |  |  |  |  |  | rs9190576 |  | |
| 15_21349904_G/T | T | RPGRIP1 | INTRONIC |  |  |  |  |  | rs22406170 | | |
| 15_21350181_T/C | C | RPGRIP1 | INTRONIC |  |  |  |  |  | rs22406174 | | |
| 15_21350636_G/A | A | RPGRIP1 | INTRONIC |  |  |  |  |  | rs22406186 | | |
| 15_21350700_T/C | C | RPGRIP1 | INTRONIC |  |  |  |  |  | rs22406187 | | |
| 15_21351375_A/G | G | RPGRIP1 | INTRONIC |  |  |  |  |  | rs8849913 |  | |
| 15_21357000_A/G | G | RPGRIP1 | INTRONIC |  |  |  |  |  | rs22406197 | | |
| 15_21359338_G/C | C | RPGRIP1 | INTRONIC |  |  |  |  |  | - |  | |
| 15_21360274_C/T | T | RPGRIP1 | INTRONIC |  |  |  |  |  | rs22406218 | | |
| 15_21360585_A/G | G | RPGRIP1 | INTRONIC |  |  |  |  |  | rs22406225 | | |
| 15_21361050_G/A | A | RPGRIP1 | INTRONIC |  |  |  |  |  | rs22406237 | | |
| 15_21361289_T/C | C | RPGRIP1 | INTRONIC |  |  |  |  |  | - |  | |
| 15_21363655_C/T | T | RPGRIP1 | INTRONIC |  |  |  |  |  | rs8798989 |  | |
| 15_21363729_G/A | A | RPGRIP1 | INTRONIC |  |  |  |  |  | rs8798990 |  | |
| 15_21364923_G/A | A | RPGRIP1 | NON_SYNONYMOUS_CODING | 2668 | 2668 | 890 | E/K | Gag/Aag | rs22406240 | | |
| 15_21365046_T/C | C | RPGRIP1 | INTRONIC |  |  |  |  |  | rs22406241 | | |
| 15_21365203_A/G | G | RPGRIP1 | SYNONYMOUS_CODING | 2877 | 2877 | 959 | T | acA/acG | - |  | |
| 15_21365993_C/A | A | RPGRIP1 | INTRONIC |  |  |  |  |  | - |  | |
| 15_21367976_A/T | T | RPGRIP1 | INTRONIC |  |  |  |  |  | rs22406243 | | |
| 15_21372351_G/A | A | RPGRIP1 | INTRONIC |  |  |  |  |  | rs22406263 | | |
| 15_21379705_C/G | G | RPGRIP1 | INTRONIC |  |  |  |  |  | - |  | |
| 15_21379706_T/A | A | RPGRIP1 | INTRONIC |  |  |  |  |  | - |  | |
| 15_21379898_G/A | A | RPGRIP1 | INTRONIC |  |  |  |  |  | rs22406266 | | |
| 15_21379926_C/T | T | RPGRIP1 | INTRONIC |  |  |  |  |  | - |  | |
| 15_21379932_C/T | T | RPGRIP1 | INTRONIC |  |  |  |  |  | - |  | |
| 15_21381091_C/T | T | RPGRIP1 | INTRONIC |  |  |  |  |  | rs22418872 | | |
| 15_21381574_C/T | T | RPGRIP1 | INTRONIC |  |  |  |  |  | - |  | |
| 15_21381891_T/C | C | RPGRIP1 | INTRONIC |  |  |  |  |  | rs22418875 | | |
| 15_21393086_C/G | G | RPGRIP1 | INTRONIC |  |  |  |  |  | - |  | |
| 15_21407892_C/T | T | SUPT16H suppressor of Ty 16 homolog | INTRONIC |  |  |  |  |  | - | Component of FACT complex (Facilitates active chromatin transcription) | |
| 15_21409296_A/G | G | SUPT16H | INTRONIC |  |  |  |  |  | rs22418886 | | |
| 15_21415013_G/A | A | CHD8  chromodomain helicase DNA binding protein 8/ SUPTH16 | INTRONIC |  |  |  |  |  | - | Chromatin remodelling factor, negative regulator of transcription | |
| 15_21426550_A/T | T | CHD8 | INTRONIC |  |  |  |  |  |  |  | |
| 15_21426580_A/T | T | CHD8 | INTRONIC |  |  |  |  |  |  |  | |
| 15_21426581_G/A | A | CHD8 | INTRONIC |  |  |  |  |  |  |  | |
| 15_21426603_T/A | A | CHD8 | INTRONIC |  |  |  |  |  |  |  | |
| 15_21426604_C/T | T | SUPT16H | INTRONIC |  |  |  |  |  |  |  | |
| 15_21426609_G/T | T | CHD8 | INTRONIC |  |  |  |  |  |  |  | |
| 15_21432109_C/T | T | CHD8 | INTRONIC |  |  |  |  |  | rs9130273 |  | |
| 15_21444288_A/G | G | CHD8 | NON_SYNONYMOUS_CODING | 6091 | 6091 | 2031 | F/L | Ttc/Ctc | rs22418887 | | |
| 15_21444288_A/G | G | snoRNA | UPSTREAM |  |  |  |  |  | rs22418887 | | |
| 15_21481126_A/G | G | CHD8 | UPSTREAM |  |  |  |  |  | - |  | |
| 15_21482931_A/C | C | CHD8 | UPSTREAM |  |  |  |  |  | - |  | |
| 15_21482932_C/A | A | CHD8 | UPSTREAM |  |  |  |  |  | - |  | |
| 15_21482970_A/C | C | CHD8 | UPSTREAM |  |  |  |  |  | - |  | |
| 15_21482971_C/T | T | CHD8 | UPSTREAM |  |  |  |  |  | - |  | |
| 15_21483059_C/T | T | CHD8 | UPSTREAM |  |  |  |  |  | - |  | |
| 15_21483061_G/C | C | CHD8 | UPSTREAM |  |  |  |  |  | - |  | |
| 15_21483065_G/C | C | CHD8 | UPSTREAM |  |  |  |  |  | - |  | |
| 15_21483071_T/C | C | CHD8 | UPSTREAM |  |  |  |  |  | - |  | |
| 15_21483098_G/T | T | CHD8 | UPSTREAM |  |  |  |  |  | - |  | |
| 15_21500599_A/G | G | RAB2B member Ras oncogene family | DOWNSTREAM |  |  |  |  |  | rs22430224 | | |
| 15_21500862_C/G | G | RAB2B | DOWNSTREAM |  |  |  |  |  | - |  | |
| 15_21501714_A/G | G | RAB2B | DOWNSTREAM |  |  |  |  |  | - |  | |
| 15_21502563_C/G | G | RAB2B | DOWNSTREAM |  |  |  |  |  | - |  | |
| 15_21502921_T/A | A | RAB2B | INTRONIC |  |  |  |  |  | - |  | |
| 15_21504942_T/G | G | RAB2B | INTRONIC |  |  |  |  |  | - |  | |
| 15_21505866_T/A | A | RAB2B | INTRONIC |  |  |  |  |  | - |  | |
| 15_21506670_G/A | A | RAB2B | INTRONIC |  |  |  |  |  | - |  | |
| 15_21507038_C/G | G | RAB2B | INTRONIC |  |  |  |  |  | - |  | |
| 15_21507981_C/T | T | RAB2B | INTRONIC |  |  |  |  |  | - |  | |
| 15_21508995_C/T | T | RAB2B | INTRONIC |  |  |  |  |  | - |  | |
| 15_21513905_C/G | G | RAB2B | INTRONIC |  |  |  |  |  | - |  | |
| 15_21515323_C/T | T | TOX4 TOX HMG box family member 4 | UPSTREAM |  |  |  |  |  | - | Component of the PTW/PP1 phosphatase complex,with role in the control of chromatin structure. Universal expression | |
| 15_21516069_G/A | A | RAB2B | INTRONIC |  |  |  |  |  | - |  | |
| 15_21516345_G/A | A | RAB2B | INTRONIC |  |  |  |  |  | - |  | |
| 15_21517124_A/G | G | RAB2B | INTRONIC |  |  |  |  |  | - |  | |
| 15_21517906_A/T | T | RAB2B | INTRONIC |  |  |  |  |  | - |  | |
| 15_21520464_C/T | T | RAB2B | UPSTREAM |  |  |  |  |  | - |  | |
| 15_21520577_A/T | T | TOX4 | INTRONIC |  |  |  |  |  | - |  | |
| 15_21522466_C/T | T | RAB2B | UPSTREAM |  |  |  |  |  | - |  | |
| 15_21522474_G/T | T | TOX4 | INTRONIC |  |  |  |  |  | - |  | |
| 15_21522476_G/T | T | RAB2B | UPSTREAM |  |  |  |  |  | - |  | |
| 15_21522480_C/T | T | TOX4 | INTRONIC |  |  |  |  |  | - |  | |
| 15_21522924_A/T | T | RAB2B | UPSTREAM |  |  |  |  |  | - |  | |
| 15_21522928_A/T | T | TOX4 | INTRONIC |  |  |  |  |  | - |  | |
| 15_21523066_A/T | T | RAB2B | UPSTREAM |  |  |  |  |  | - |  | |
| 15_21524966_C/T | T | TOX4 | INTRONIC |  |  |  |  |  | - |  | |
| 15_21528657_A/G | G | TOX4 | INTRONIC |  |  |  |  |  | - |  | |
| 15_21530468_G/T | T | TOX4 | INTRONIC |  |  |  |  |  | rs22418890 | | |
| 15_21531948_A/C | C | TOX4 | SYNONYMOUS_CODING | 1005 | 1005 | 335 | A | gcA/gcC | - |  | |
| 15_21535187_T/C | C | TOX4 | DOWNSTREAM |  |  |  |  |  | rs22380363 | | |
| 15_21541583_C/G | G | METTL3 methyltransferase like 3 | INTRONIC |  |  |  |  |  | - | N6 adenosine methyl transferase | |
| 15_21541627_C/T | T | METTL3 | INTRONIC |  |  |  |  |  | - |  | |
| 15_21541639_A/T | T | METTL3 | INTRONIC |  |  |  |  |  | - |  | |
| 15_21541680_G/A | A | METTL3 | INTRONIC |  |  |  |  |  | - |  | |
| 15_21541685_G/A | A | METTL3 | INTRONIC |  |  |  |  |  | - |  | |
| 15_21543671_A/T | T | METTL3 | INTRONIC |  |  |  |  |  | - |  | |
| 15_21544881_G/T | T | METTL3 | INTRONIC |  |  |  |  |  | - |  | |
| 15_21545344_T/C | C | METTL3 | INTRONIC |  |  |  |  |  | - |  | |
| 15_21545345_G/A | A | METTL3 | INTRONIC |  |  |  |  |  | - |  | |
| 15_21545347_G/A | A | METTL3 | INTRONIC |  |  |  |  |  | - |  | |
| 15_21545553_A/G | G | METTL3 | INTRONIC |  |  |  |  |  | - |  | |
| 15_21545892_A/G | G | METTL3 | INTRONIC |  |  |  |  |  | - |  | |
| 15_21550327_G/T | T | METTL3 | INTRONIC |  |  |  |  |  | - |  | |
| 15_21550334_C/T | T | METTL3 | INTRONIC |  |  |  |  |  | - |  | |
| 15_21550336_T/C | C | METTL3 | INTRONIC |  |  |  |  |  | - |  | |
| 15_21550423_C/T | T | METTL3 | INTRONIC |  |  |  |  |  | - |  | |
| 15_21550425_T/C | C | METTL3 | INTRONIC |  |  |  |  |  | - |  | |
| 15_21553115_C/G | G | METTL3 | UPSTREAM |  |  |  |  |  | - |  | |
| 15_21555188_G/A | A | METTL3 | UPSTREAM |  |  |  |  |  | - |  | |
| 15_21556330_A/G | G | METTL3 | UPSTREAM |  |  |  |  |  | - |  | |
| 15_21556610_G/A | A | METTL3 | UPSTREAM |  |  |  |  |  | - |  | |
| 15_21556920_G/A | A | METTL3 | UPSTREAM |  |  |  |  |  | - |  | |
| 15_21556955_T/C | C | METTL3 | UPSTREAM |  |  |  |  |  | - |  | |
| 15_21557556_A/G | G | METTL3 | UPSTREAM |  |  |  |  |  | - |  | |
| 15_21560852_C/T | T | SALL2 sal-like 2 (Drosophila) | DOWNSTREAM |  |  |  |  |  | - | Transcription factor, growth arrest functions, some expression in eye and in neural tissues | |
| 15_21561022_T/C | C | SALL2 | DOWNSTREAM |  |  |  |  |  | - |  | |
| 15_21561254_A/G | G | SALL2 | DOWNSTREAM |  |  |  |  |  | - |  | |
| 15_21561583_T/A | A | SALL2 | DOWNSTREAM |  |  |  |  |  | - |  | |
| 15_21561585_A/T | T | SALL2 | DOWNSTREAM |  |  |  |  |  | - |  | |
| 15_21561605_T/A | A | SALL2 | DOWNSTREAM |  |  |  |  |  | - |  | |
| 15_21561607_A/T | T | SALL2 | DOWNSTREAM |  |  |  |  |  | - |  | |
| 15_21562023_A/T | T | SALL2 | DOWNSTREAM |  |  |  |  |  | - |  | |
| 15_21562024_A/T | T | SALL2 | DOWNSTREAM |  |  |  |  |  | - |  | |
| 15_21562173_C/T | T | SALL2 | DOWNSTREAM |  |  |  |  |  | - |  | |
| 15_21562858_C/T | T | SALL2 | DOWNSTREAM |  |  |  |  |  | - |  | |
| 15_21562884_T/A | A | SALL2 | DOWNSTREAM |  |  |  |  |  | - |  | |
| 15_21563048_G/A | A | SALL2 | DOWNSTREAM |  |  |  |  |  | - |  | |
| 15_21563145_A/G | G | SALL2 | DOWNSTREAM |  |  |  |  |  | - |  | |
| 15_21563339_A/G | G | SALL2 | DOWNSTREAM |  |  |  |  |  | - |  | |
| 15_21564556_A/G | G | SALL2 | DOWNSTREAM |  |  |  |  |  | - |  | |
| 15_21565732_A/C | C | SALL2 | NON_SYNONYMOUS_CODING | 2290 | 2290 | 764 | S/A | Tca/Gca | - |  | |
| 15_21565733_A/G | G | SALL2 | SYNONYMOUS_CODING | 2289 | 2289 | 763 | C | tgT/tgC | - |  | |
| 15_21565736_A/G | G | SALL2 | SYNONYMOUS_CODING | 2286 | 2286 | 762 | C | tgT/tgC | - |  | |
| 15_21566495_A/G | G | SALL2 | SYNONYMOUS_CODING | 1764 | 1764 | 588 | P | ccT/ccC | - |  | |
| 15_21567695_G/C | C | SALL2 | SYNONYMOUS_CODING | 678 | 678 | 226 | S | tcC/tcG | - |  | |
