# Supplementary material for: Multiple Mechanisms Contribute to Leakiness of a Frameshift Mutation in Canine Cone-Rod Dystrophy
Source: PLoS One. 2012 Dec 12;7(12):e51598. doi: 10.1371/journal.pone.0051598 (PMC3520932; doi:10.1371/journal.pone.0051598)
Supplement: Table S2 — Transcripts in Genbank with equivalent splice patterns to those reported here. (DOC) [file pone.0051598.s003.doc]

**Table S2**

| This M/S | Genbank Canine Full Length Transcript (putative) | Genbank Canine spliced EST (putative) | Genbank non-canine Full Length Transcript (putative) |
| --- | --- | --- | --- |
| CR1^1^ |  |  |  |
| CR2 |  |  | Bos AF265669 |
| CR3 | HMG021768* |  |  |
| CR4 | HMG021768* |  |  |
| CR5 | HMG021768* |  |  |
| CR6 | HMG021768* |  |  |
| CR7 |  | BM536941, BM537079 |  |
| CR8^2^ |  |  | Bos AF265669 |
| CR9^1^ |  |  |  |
| CR10 | HMG021769, HMG021770, HMG021771 |  |  |
| CR11^2^ |  |  | Mus AY008297, other murine forms |
| CR12^2^ |  |  | Mac AB169288 |
| CR13 | HMG021768, HMG021769, HMG021770, HMG021771 | HQ315863, HQ315864 |  |
| CR14 | HMG021769 | BM536941, BM537079 |  |
| CR15 |  | HQ315863 |  |
| CR16 | HMG021768 | Many |  |

Footnotes: Superscript ^1^: No exact equivalent reported in any species

Superscript ^2^: equivalents reported in other species but not in dog.

*: Truncated at 5’ end in this M/S.
