# Supplementary material for: Multiple Mechanisms Contribute to Leakiness of a Frameshift Mutation in Canine Cone-Rod Dystrophy
Source: PLoS One. 2012 Dec 12;7(12):e51598. doi: 10.1371/journal.pone.0051598 (PMC3520932; doi:10.1371/journal.pone.0051598)
Supplement: Table S3 — Primers used in cDNA analysis and for qRT-PCR. (DOC) [file pone.0051598.s004.doc]

**Table S3**

| **Primer #** | **Exon** | **Orientation** | **Sequence (5’>3’)** | **T_m_ (**°C**)** | **Application** |
| --- | --- | --- | --- | --- | --- |
| 2921 | 0 | F | GCGTACAGCGCATCAAAGGAC | 63 | RT-PCR |
| 2209 | 7 | F | CATTAGTAGCAACAAAAGCACA | 56 | RT-PCR |
| 2663 | 14 | F | CCCTGCAGCGAGCCATCA | 61 | RT-PCR |
| 2588 | 15 | F | AAGCCGCGGCCTGCCACT | 63 | RT-PCR |
| 2563 | 1 | R | CAGGGATCCTTTTGATCTCATCCTGTTGCT |  | 5’-RACE |
| 2208 | 9 | R | TGGGCAGCACTCAGGATTC | 60 | 5’-RACE |
| 2587 | 12 | R | TCCCCCACGAGCTCACTG | 61 | 5’-RACE |
| 2219 | 14 | R | CTGGTAACACACGTTGATGG | 58 | 5’-RACE |
| 2589 | 15 | R | TCGTCATCACTGCTTCCAGT | 58 | RT-PCR, 5’-RACE |
| 2666 | 19 | R | CCGCAGGGTCAGTGAGGT | 61 | RT-PCR |
| 2223 | 26 | R | CGTCGTCCAGAGGATCACTT | 60 | RT-PCR, 5’-RACE |
| 2664 | qX16R | R | GAGCTGGCTGTGTTTCCAG | 60 | QRT-PCR |
| 2994 | qX15F | F | GACGAAAGCAGACAATGACAGT | 60 | QRT-PCR |
| 2995 | qX15B/19R | R | AGTGAGGTTAAAATCACCGGGT | 60 | QRT-PCR |
| 2999 | qX18F | F | TCTGACCTGGATAAATATCTGAG | 59 | QRT-PCR |
| 3000 | qX18/19R | R | GTCAGTGAGGTTAAAATCACCTTTA | 61 | QRT-PCR |
| 3003 | qX21F | F | GGAAAGGGAACATCAGGTTGC | 61 | QRT-PCR |
| 3004 | qX21/22R | R | ATTCAGTGTAGTTCACCTGTTGTA | 60 | QRT-PCR |
| 3005 | qX0/1F | F | GTACCAGTGTCGAAAGGTAAG | 60 | QRT-PCR |
| 3006 | qX1R | R | TCCAAGAAAGCTCCTTCACCA | 60 | QRT-PCR |
| 3019 | qX14/15F | F | CAACGTGTGTTACCAGGAGGA | 61 | QRT-PCR |
| 3020 | qX15R | R | AGCTGACGGATGCGGTGG | 61 | QRT-PCR |
| 3021 | qX23F | F | TTCTGAAGCCAGTGAAGCACA | 60 | QRT-PCR |
| 3022 | qX23/24R | R | CATCTTCTCCGAGTCTGCCT | 61 | QRT-PCR |
| 3023 | qX25F | F | CCGAAGGCAGTTCCTGCG | 61 | QRT-PCR |
| 3024 | qX26R | R | ACATTCCTTTTCGTCGTCCAG | 60 | QRT-PCR |
| 3038 | qX10F | F | CAGGAGAGAGTTGAAGATTTGGA | 61 | QRT-PCR |
| 3039 | qX12R | R | TGAGGCTGATTACTGCTGGC | 61 | QRT-PCR |
