# Supplementary material for: Multiple Mechanisms Contribute to Leakiness of a Frameshift Mutation in Canine Cone-Rod Dystrophy
Source: PLoS One. 2012 Dec 12;7(12):e51598. doi: 10.1371/journal.pone.0051598 (PMC3520932; doi:10.1371/journal.pone.0051598)
Supplement: Table S4 — Primers used for microsatellite genotyping. These primers were used in checking haplotypes around RPGRIP1. (DOC) [file pone.0051598.s005.doc]

Table S4

| **Feature** | **CFA15 location (Mb)** | **Forward (5’>3’)** | **Reverse (5’>3’)** |
| --- | --- | --- | --- |
| **CAMC15.036** | 17.32 | ACATTGGGTTCCGCATTCAGT | CCTGTGTGGCAGCAGTTGAAT |
| **CAMC15.037** | 18.13 | TGGCGCGGTGGTTTAGCAT | TTTCCAACCTCCCTCCAACC |
| **CAMC15.038** | 18.76 | TCCTCGTCTCTACAGTGGGCT | GCTACATGTCAGGCGTTGTGT |
| **CAMC15.039** | 19.87 | CATCATCGGGGAAACCCAAGT | AACGCTTAGCTTGCTTCCACG |
| **CAMC15.040** | 20.39 | TCCCCCATTATTGGATGGCCT | ATGGTGGTAATCACGGTGCAA |
| **CAMC15.041** | 21.05 | AGCTGTTCTGTGGGGTGCTA | CAGGTGTGAGTTGTGGGTCTT |
