# Supplementary material for: Multiple Mechanisms Contribute to Leakiness of a Frameshift Mutation in Canine Cone-Rod Dystrophy
Source: PLoS One. 2012 Dec 12;7(12):e51598. doi: 10.1371/journal.pone.0051598 (PMC3520932; doi:10.1371/journal.pone.0051598)
Supplement: Table S5 — Primers used for analyzing the polyA tract and for constructing plasmids for dual-reporter assay. (DOC) [file pone.0051598.s006.doc]

Table S5

| **Primer#** | **Feature** | **Purpose** | **Orientation** | **Sequence** |
| --- | --- | --- | --- | --- |
| **2561** | *RPGRIP1*, exon2 | p2luc/oligoA plasmid | F | CAGGTCGACGGTGAAGGAGCTTTCTTGGA |
| **2563** | *RPGRIP1*, exon2 | p2luc/oligoA and p2luc/R- plasmids | R | CAGGGATCCTTTTGATCTCATCCTGTTGCT |
| **2578** | p2luc | p2luc insertion sequencing | F | GCAAGAAGATGCACCTGATG |
| **2579** | p2luc | p2luc insertion sequencing | R | CCGGGCCTTTCTTTATGTTTT |
| **2742** | *RPGRIP1*, exon2 | P2luc/R+ plasmid | F | TGCAGTCGACGGAAGAATTGGAGGACAGCT |
| **2743** | *RPGRIP1*, exon2 | P2luc/R+ plasmid | R | TGCAGGATCCGATCTCATCCTGTTGCTTCC |
| **2671** | *RPGRIP1*, exon2 | P2luc/R- plasmid | F | CAGTGTCGACGAATTGGAGGACAGCTTGTTT |
| **2181** | *RPGRIP1*, exon2 | OligoA sizing/sequencing, Genomic clone sequencing | F | GAAGAGCACATGTTGGTGAAGG |
| **2189** | *RPGRIP1*, intron2 | OligoA sizing/sequencing | R | CTTAAGGAGAACACAAGGTAC |
| **2229** | *RPGRIP1*, intron1 | Colony screening | F | GGCTGCAGTGGTTCTGAGAC |
| **2230** | *RPGRIP1*, intron1 | Colony screening | R | TCAAGGGTGGTTGAGCTTTC |
